# Microbial functional gene assembly is associated with soil carbon and nitrogen dynamics during grassland degradation

**DOI:** 10.3389/fmicb.2026.1878594

**Published:** 2026-07-08

**Authors:** Zhennan He, Rui Hua, Tiecheng Wu, Hui Qu, Guolin Yang, Siyi Wang, Fengqin Gao, Yuanyuan Jing

**Affiliations:** 1 Grassland Research Institute, CAAS, Hohhot, Inner Mongolia, China; 2 College of Grassland Science, Qingdao Agricultural University, Chengyang Campus, Qingdao, Shandong, China; 3 Inner Mongolia Academy of Agricultural & Animal Husbandry Sciences, Hohhot, Inner Mongolia, China

**Keywords:** carbon and nitrogen cycling, functional genes, grassland degradation, microbial biomass carbon, microbial community assembly, mineral-associated organic carbon

## Abstract

**Introduction:**

Grassland degradation is often accompanied by changes in the structure and function of soil microbial communities. However, the mechanisms by which the assembly of microbial functional communities is associated with alterations in soil carbon and nitrogen pools remain unclear.

**Methods:**

This study was conducted along a degradation gradient in a typical steppe of Inner Mongolia. Metagenomics, community null models, and structural equation modeling were used to examine microbial functional gene assembly, carbon and nitrogen cycling genes, and their associations with soil carbon and nitrogen pools.

**Results:**

The assembly of microbial functions shifted from being predominantly influenced by stochastic processes to deterministic processes, with the strongest deterministic filtering observed during the moderate degradation stage. The abundance of the aerobic oxidation gene porA decreased with increasing degradation, whereas fermentation genes, including ldh and atoB, increased significantly during moderate degradation. Denitrification genes, including narG, nirK, norB, and nosZ, reached their highest abundance during the heavy degradation stage. Mineral-associated organic carbon exhibited a nonlinear pattern characterized by an initial increase followed by a decrease. Structural equation modeling revealed that microbial biomass carbon was the central variable linking microbial functional differentiation with changes in soil carbon and nitrogen pools. During the heavy degradation stage, soil ammonium nitrogen showed a numerical increase, suggesting that nitrogen released from mineral-associated organic carbon decomposition may be predominantly converted into inorganic forms.

**Discussion:**

These findings indicate that the threshold-like decline of microbial biomass carbon, rather than specific restructuring of functional gene profiles, was closely associated with the collapse of stable carbon-nitrogen pool stability during grassland degradation. Changes in functional genes may therefore represent responsive signals accompanying microbial biomass carbon attenuation. The continuous decrease in microbial biomass carbon and associated shifts in functional gene ratios may serve as potential indicators of declining carbon-nitrogen stability in grassland soils.

## 1 Introduction

Grassland ecosystems serve as a cornerstone for global livestock production and ecological security, making their sustainable management crucial for maintaining livelihoods and ecological balance (Guo et al., 2024; Liu et al., 2025). However, irrational utilization and climate change have led to widespread grassland degradation, which poses a global threat to their ecological functions and services (Ohlert et al., 2025). Against this backdrop, a persistent challenge that has perplexed managers and researchers is that, even under uniform stocking rate standards, grasslands often develop highly heterogeneous degradation patches. Factors such as selective grazing by livestock, pest interference, and uneven distribution of vegetation species collectively contribute to significant spatial variability in pasture productivity. This renders “one-size-fits-all” management policies not only ineffective but also potentially accelerates localized ecological collapse (Dong et al., 2025; Teague and Dowhower, 2003). This prevalent management dilemma suggests that the key factors related to differential degradation may not be the external pressures themselves, but rather the nonlinear responses of intrinsic ecosystem properties to these external stresses (Gao et al., 2025).

Currently, the assessment and monitoring of grassland degradation primarily rely on apparent static indicators such as vegetation coverage and aboveground biomass (Xu et al., 2025; Yan et al., 2021). Although these metrics are easy to monitor, they essentially represent the “outcome” rather than “preceding indicators” of ecosystem decline, making it challenging to analyze the underground biological processes associated with fundamental system shifts. Soil microorganisms, as core drivers of underground carbon-nitrogen cycles and key regulators of soil health, are closely linked to degradation processes through their community and functional responses.

In recent years, important advances have been made in understanding soil microbial communities in degraded grasslands. Regarding community structure and function, numerous studies have documented changes in microbial community composition during degradation, revealing differential responses of bacterial and fungal communities (Zhang et al., 2020; Shi et al., 2025; Zheng et al., 2021). In terms of community assembly mechanisms, microbial assembly processes can shift from stochastic to deterministic dominance along grazing or degradation gradients, with bacteria and fungi often exhibiting decoupled patterns (Liu N. et al., 2021; Julihaiti et al., 2025). With respect to functional genes, metagenomic studies have revealed significant effects of disturbance on the abundance of carbon and nitrogen cycling genes (Hu et al., 2025; Wang C. et al., 2025). Regarding soil carbon pool dynamics, the nonlinear responses of mineral-associated organic carbon (MAOC) to environmental gradients and their regulatory factors have been preliminarily quantified (Wang G. et al., 2025; Yang et al., 2025), and microbial biomass carbon (MBC) has been widely recognized as a sensitive indicator of changes in soil quality (Blagodatskaya et al., 2026).

Despite these advances, three critical knowledge gaps remain. First, community assembly studies have predominantly focused on the taxonomic level, leaving the assembly processes at the functional gene level and their quantitative shifts along degradation gradients poorly understood. Second, most functional gene studies have concentrated on a limited number of genes within single cycling pathways (Chai et al., 2025), lacking systematic analysis of carbon and nitrogen metabolic networks and consequently lacking mechanistic understanding of the synchrony in carbon and nitrogen cycling changes. Third, current studies generally discuss community assembly (primarily based on taxonomic diversity) and community function (focused on a few discrete functional genes) on separate tracks, failing to reveal how functional gene communities reconfigure their metabolic networks during shifts in assembly mechanisms, and unable to establish quantitative associations from microbial functional responses to changes in soil carbon and nitrogen pools. This disconnection along the “assembly–function–carbon pool” chain hinders the development of microbiological indicators capable of quantitatively diagnosing system states and supporting process-based ecosystem management. Revealing the assembly mechanisms of microbial functional communities along degradation gradients and their associations with soil carbon and nitrogen pools constitutes a key scientific question in grassland ecology.

This study established a transect along a gradient of grassland degradation under a unified stocking rate management regime, integrating metagenomics, community null model analysis, and SEM. We focused on the most stable carbon fraction in soil—mineral-associated organic carbon (MAOC)—as its nonlinear dynamics may indicate critical transitions in system states. Additionally, total nitrogen (TN) was incorporated into the core analytical framework to examine whether carbon and nitrogen pools achieve synchronous changes through the pivotal role of MAOC. We aim to address three interconnected scientific questions: (1) How does the assembly process of microbial functional gene communities undergo quantitative shifts along the gradients of grassland degradation succession? (2) Does this shift coincide with systematic changes in carbon and nitrogen metabolic gene profiles? (3) Is there an indirect quantitative association between the restructuring of microbial functional modules and changes in MAOC and TN, and does microbial biomass carbon (MBC) play a central role in this process?

Based on this, we propose the following hypotheses: (1) As degradation intensifies, the assembly process of microbial functional communities transitions from being predominantly stochastic to deterministic; (2) This shift in assembly mechanisms is accompanied by a reconfiguration of carbon and nitrogen metabolic gene profiles, characterized by a decrease in aerobic oxidation-related genes, an increase in fermentation and maintenance metabolism-related genes, a heightened abundance of denitrification-related genes during severe degradation stages, and a negative covariance between carbon-energy regulation modules and denitrification functional modules; (3) The degradation of soil physical properties may affect soil carbon and nitrogen pools indirectly through associations with microbial biomass carbon (MBC), with the threshold-like decline in MBC serving as a central hub linking functional differentiation to changes in MAOC and TN. Functional gene profile shifts are more likely a concomitant response to the decline in MBC rather than an independent direct factor. A close association may exist between the disintegration of MAOC and the decline of TN. By testing these hypotheses, we aim to identify microbial functional response patterns associated with degradation gradients, thereby providing a potential biological reference for grassland health assessment and zonal management.

## 2 Materials and methods

### 2.1 Overview of the study area

The study area is situated in the core region of the Xilingol typical steppe in Inner Mongolia, which constitutes a significant component of the Eurasian grassland ecosystem. This region is characterized by a temperate continental monsoon climate, with an annual average temperature ranging from 0 to 3 °C and annual precipitation between 200 and 350 mm. The zonal vegetation is primarily composed of *Leymus chinensis*, *Stipa krylovii*, and *Cleistogenes squarrosa*. All study sites were located on independent pastures that have been continuously managed by fixed herding households since the grassland tenure was established in the 1980s. According to household surveys, the grazing pressure of each pasture has remained relatively stable over the past decade, with an average stocking rate of approximately 14.5 sheep units per hectare. Pastures that had experienced fire, cultivation, or enclosure changes within the past 5 years were excluded.

### 2.2 Experimental design and sampling

To analyze the underground mechanisms of heterogeneous grassland degradation, this study employed a “space-for-time” research design. We established a clear four-level degradation gradient based on the National Standard of the People’s Republic of China, “Classification Indexes for Degradation, Sandification and Salinization of Natural Grasslands” (GB 19377-2003), supplemented by field vegetation surveys. This standard integrates vegetation coverage, aboveground biomass, and the proportion of palatable forage, thereby reflecting the comprehensive degradation status of each pasture rather than a single degradation cause. All sampling activities were completed in August 2024.

To maximize the validity of the space-for-time assumption, the following measures were implemented in site selection and sampling design:

1. *Independent replicates and spatial independence*: Each degradation level included six spatially independent pastures, with a minimum distance of 2 km between pastures. The six pastures within each degradation level were spatially interspersed rather than clustered in a single area, thereby avoiding the pseudo-replication problem defined by Hurlbert (1984)—the use of inferential statistics to test treatment effects with replicates that are not statistically independent. Within each pasture, sampling points were arranged in a “*Z*” pattern, with adjacent points spaced at least 50 m apart. Soil samples were collected from a depth of 0–10 cm using a 5 cm diameter soil auger, with visible plant residues removed prior to collection. Samples from five points within the same pasture were thoroughly mixed to form one composite sample. Six independent biological replicates were thus established for each degradation level, corresponding to the six independent pastures.
2. *Control of spatial autocorrelation*: Spatial autocorrelation is a common phenomenon. To minimize its potential interference, our sampling intervals (≥50 m within pastures, >2 km between pastures) substantially exceeded the effective autocorrelation range of soil properties reported for typical steppe grasslands. Ritz et al. (2004) demonstrated through geostatistical analysis that the autocorrelation ranges of most soil chemical and microbiological properties in grazed grasslands are between 0.6 and 6 m.
3. *Clarification of research objectives*: Since the specific degradation trajectories of individual pastures (e.g., fluctuations in grazing intensity, rodent damage, climate variability) may differ and cannot be fully reconstructed, the objective of this study is not to strictly demonstrate a causal succession sequence from ND to HD, but rather to compare systematic differences in soil properties and microbial communities across distinct degradation states.
4. *Sample collection and preservation*: Each collected soil sample was promptly divided into two portions after mixing: one portion was placed in a sterile centrifuge tube, immediately frozen in liquid nitrogen, and subsequently transferred to a −80 °C ultra-low temperature freezer for storage, intended for future metagenomic DNA extraction. The other portion was placed in a ziplock bag and stored in an ice box. Fresh soil samples from this portion were later analyzed in the laboratory to measure MBC, microbial biomass nitrogen (MBN), and various soil physicochemical properties. Concurrently, aboveground plant biomass (PB) surveys were conducted by cutting the plants at ground level, followed by drying the samples in a 65 °C oven until reaching a constant weight before weighing. The soil physicochemical data and PB data from this study are presented in Supplementary Tables 1 and 2.

Soil physicochemical properties were determined following Bao (2000) and Cambardella and Elliott (1992). Soil water content (SWC) was determined by oven-drying at 105 °C for 24 h. Soil bulk density (Sd) was measured using the cutting ring method. Soil aggregates were separated by dry sieving, and the mass percentages of macroaggregates (>0.25 mm, MacA) and microaggregates (<0.25 mm, MicA) were calculated. Soil organic carbon (SOC) was determined by the potassium dichromate oxidation-external heating method. Prior to determination, samples were pretreated with dilute HCl to remove inorganic carbon. Total nitrogen (TN) was measured using the Kjeldahl method (FOSS 2300 automatic nitrogen analyzer). Ammonium nitrogen (NH₄^+^-N) and nitrate nitrogen (NO₃^−^-N) were extracted with 2 M KCl solution and determined by indophenol blue colorimetry and ultraviolet spectrophotometry, respectively. Total phosphorus (TP) and available phosphorus (AP) were determined by sodium bicarbonate extraction-molybdenum antimony colorimetric method. Total potassium (TK) and available potassium (AK) were determined by ammonium acetate extraction-flame photometry. Microbial biomass carbon (MBC) and microbial biomass nitrogen (MBN) were determined by the chloroform fumigation-K_2_SO_4_ extraction method. Fresh soil samples were stored at 4 °C and fumigated immediately upon return to the laboratory. Organic carbon in the extracts was measured using a TOC analyzer (Shimadzu, Japan), and total nitrogen was determined by the Kjeldahl method. Soil carbon fractionation: Particulate organic carbon (POC) and mineral-associated organic carbon (MAOC) were separated by sodium hexametaphosphate dispersion and wet sieving. Air-dried soil (20 g, <2 mm) was mixed with 100 mL of sodium hexametaphosphate solution (5 g·L^−1^) and shaken for 18 h. The dispersed suspension was passed through a 53 μm sieve. The fraction retained on the sieve (>53 μm) was the particulate organic matter, and the fraction passing through (<53 μm) was the mineral-associated organic matter. Both fractions were dried at 60 °C to constant weight, and organic carbon content was determined by the potassium dichromate oxidation-external heating method. POC content was calculated as the organic carbon concentration in the >53 μm fraction, and MAOC content was calculated as the organic carbon concentration in the <53 μm fraction.

### 2.3 Metagenomic sequencing and bioinformatics analysis

Total genomic DNA was extracted from 0.5 g of frozen soil using the FastDNA SPIN Kit for Soil (MP Biomedicals, United States), following the manufacturer’s protocol. The concentration and purity of the DNA were assessed using a NanoDrop 2000 spectrophotometer and a Qubit 4.0 fluorometer. Qualified samples were subsequently sent to Shanghai Personal Biotechnology Co., Ltd. for paired-end sequencing (2 × 150 bp) on the Illumina NovaSeq 6,000 platform, targeting a data volume of 10 GB per sample.

The raw sequencing data underwent quality control and adapter trimming using Trimmomatic (v0.39), resulting in high-quality clean data. *De novo* assembly was conducted with MEGAHIT (v1.2.9), followed by gene prediction using MetaGeneMark (v3.38). The predicted non-redundant genes were clustered into a gene set utilizing CD-HIT (v4.8.1) with clustering parameters set at 95% identity and 90% coverage. High-quality sequences from each sample were aligned to the non-redundant gene set using SOAPaligner (v2.21) to compute gene abundance, expressed as RPKM values.

Gene function annotation was conducted using BLASTP alignment (*e*-value ≤1*e* − 5) with DIAMOND (v2.0.13), referencing the KEGG database (Release 107.0, January 2023). To systematically identify functional genes involved in carbon and nitrogen cycling, the PSN Biogeochemical Cycling functional gene set (Personalbio, Shanghai) was used. This gene set integrates multiple databases including KEGG, Metacyc, CAZy, NCyc, MCyc, and DiTing to curate genes associated with key element cycles. Carbon and nitrogen cycling genes were extracted from the annotation results and mapped to corresponding metabolic pathways (e.g., carbon fixation, glycolysis, fermentation, nitrification, and denitrification) using the integrated functional gene set. Pathway diagrams were then generated and visualized using R (v4.3.3).

### 2.4 Statistical analysis

All statistical analyses were conducted using R version 4.3.3. Differences in soil physicochemical properties and plant biomass among degradation levels were evaluated with a one-way analysis of variance (ANOVA), followed by Tukey’s honestly significant difference (HSD) *post hoc* test for all pairwise comparisons. The *β*-diversity of microbial functional gene profiles was calculated using Bray-Curtis distances and visualized through principal coordinate analysis (PCoA). The significance of intergroup differences was assessed using permutational multivariate analysis of variance (PERMANOVA) via the adonis2 function with 999 permutations. The associations between microbial functional gene profiles and individual soil physicochemical variables were evaluated using Mantel tests based on Spearman’s rank correlation with 999 permutations (vegan package, v2.6-6.1). The functional gene distance matrix was calculated using Bray–Curtis dissimilarity, and the environmental distance matrices were calculated using Euclidean distance. Sixteen environmental variables were tested individually, and Bonferroni correction was applied to adjust for multiple testing.

#### 2.4.1 Analysis of assembly mechanisms of microbial functional gene communities

To quantify the relative contributions of deterministic and stochastic processes in shaping microbial functional communities and to identify critical ecological thresholds, we performed a null model analysis based on the framework implemented in the iCAMP package (v1.5.1). The functional gene abundance profile (KO table) was used as the input community matrix. Null communities were generated using the independent swap algorithm (null.model = “independentswap”) with 999 randomizations to maintain species occurrence frequency while randomizing community composition. For each degradation level, the observed Bray–Curtis dissimilarity among samples was compared with the null distribution to calculate the normalized stochasticity ratio (NST). An NST value greater than 0.5 indicates dominance by stochastic processes, while an NST value less than 0.5 suggests dominance by deterministic processes. Additionally, the standardized effect size (SES) was calculated as (observed *β*-diversity − mean null *β*-diversity)/standard deviation of null *β*-diversity, with |SES| > 1.96 indicating significant deviation from the null expectation (*p <* 0.05).

#### 2.4.2 Structural equation modeling analysis (SEM)

Given the limited sample size (*n* = 24), we employed piecewise path analysis as an exploratory tool to examine conditional covariation patterns among variables. Accordingly, all path coefficients should be interpreted as measures of association rather than causal effects. Based on the hypotheses outlined in the Introduction, the model was structured into four hierarchical layers: (1) Plant–soil–microbe layer: We examined whether plant biomass (PB) covaried with soil water content (SWC), macroaggregates (MacA), and soil bulk density (Sd), and whether these variables showed direct covariation with MBC. (2) Microbial–functional layer: We examined whether MBC covaried with the carbon-energy regulation module (summed abundance of rbcL, bglX, amyA, and norA) and the denitrification module (summed abundance of narG, nirK, and norB), and whether the two functional modules covaried with each other. (3) Carbon pool layer: We examined whether MBC, MacA, Sd, SWC, and PB covaried with MAOC, and whether the two functional modules showed direct covariation with MAOC independent of MBC. (4) Nitrogen pool layer: We examined whether MAOC covaried with total nitrogen (TN), and whether MBC, Sd, and SWC showed direct covariation with TN independent of MAOC. An initial model including all hypothesized paths was then constructed. Linear mixed-effects models were fitted using the lme4 package, and the piecewiseSEM package was used to evaluate overall model consistency via Fisher’s C statistic, where a *p*-value greater than 0.05 indicates acceptable consistency between the model and the data. Collinearity diagnostics were conducted using variance inflation factors (VIF), with a VIF value exceeding 5 indicating severe multicollinearity. Furthermore, the stability of the path coefficients was evaluated by performing 1,000 bootstrap resampling iterations.

## 3 Results

### 3.1 Construction mechanisms of functional gene communities

**Figure 1:**
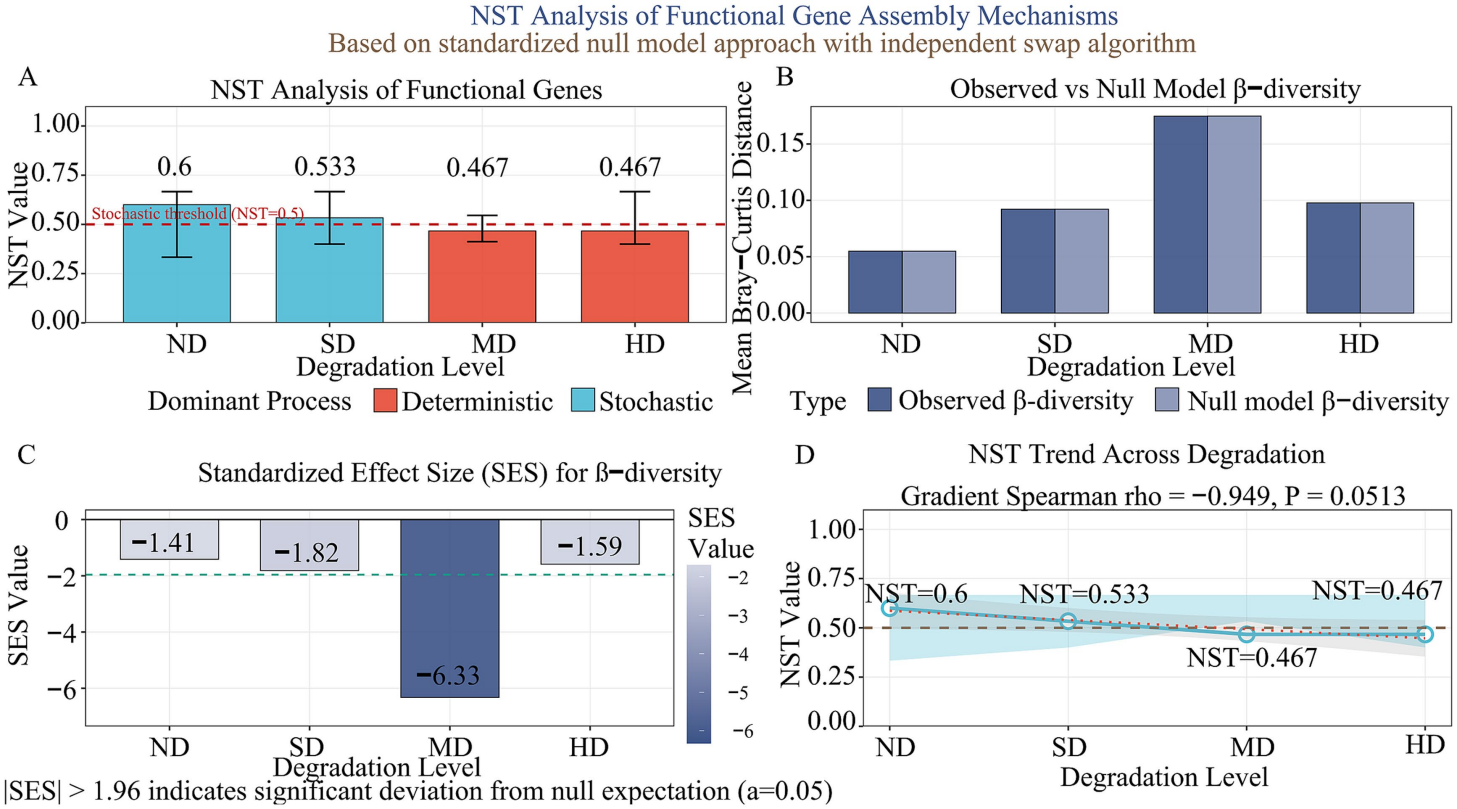
Community assembly mechanisms of microbial functional gene communities across the grassland degradation gradient. **(A)** Normalized stochasticity ratio (NST) values of functional gene communities at different degradation stages. The dashed line at NST = 0.5 indicates the threshold separating stochastic and deterministic dominance. **(B)** Comparison between observed and null-model Bray–Curtis β-diversity. **(C)** Standardized effect size (SES) of β-diversity relative to null model expectations. The dashed line indicates the significance threshold of SES = −1.96. **(D)** Trend of NST values along the degradation gradient. ND, non-degraded grassland; SD, slightly degraded grassland; MD, moderately degraded grassland; HD, heavily degraded grassland.

The null model analysis based on metagenomic KEGG Orthology (KO) functional profiles demonstrated that grassland degradation significantly influenced the assembly processes of microbial functional gene communities (Figure 1). The NST analysis revealed that the NST values for the ND and SD stages were 0.600 and 0.533, respectively, both exceeding the stochasticity threshold of 0.5. In contrast, the NST values for the MD and HD stages were 0.467, falling below 0.5. Furthermore, the NST values exhibited a negative correlation with the degradation gradient (Spearman *ρ* = −0.949, *p* = 0.051). The SES analysis further elucidated the nonlinear characteristics of the community assembly processes. The SES values for the ND, SD, and HD stages were −1.41, −1.82, and −1.59, respectively, all surpassing the significance threshold of −1.96. Notably, the SES value for the MD stage sharply decreased to −6.33, significantly deviating from the null model expectation (*p* < 0.05).

### 3.2 Overall changes in microbial functional gene profiles and their environmental drivers

**Figure 2:**
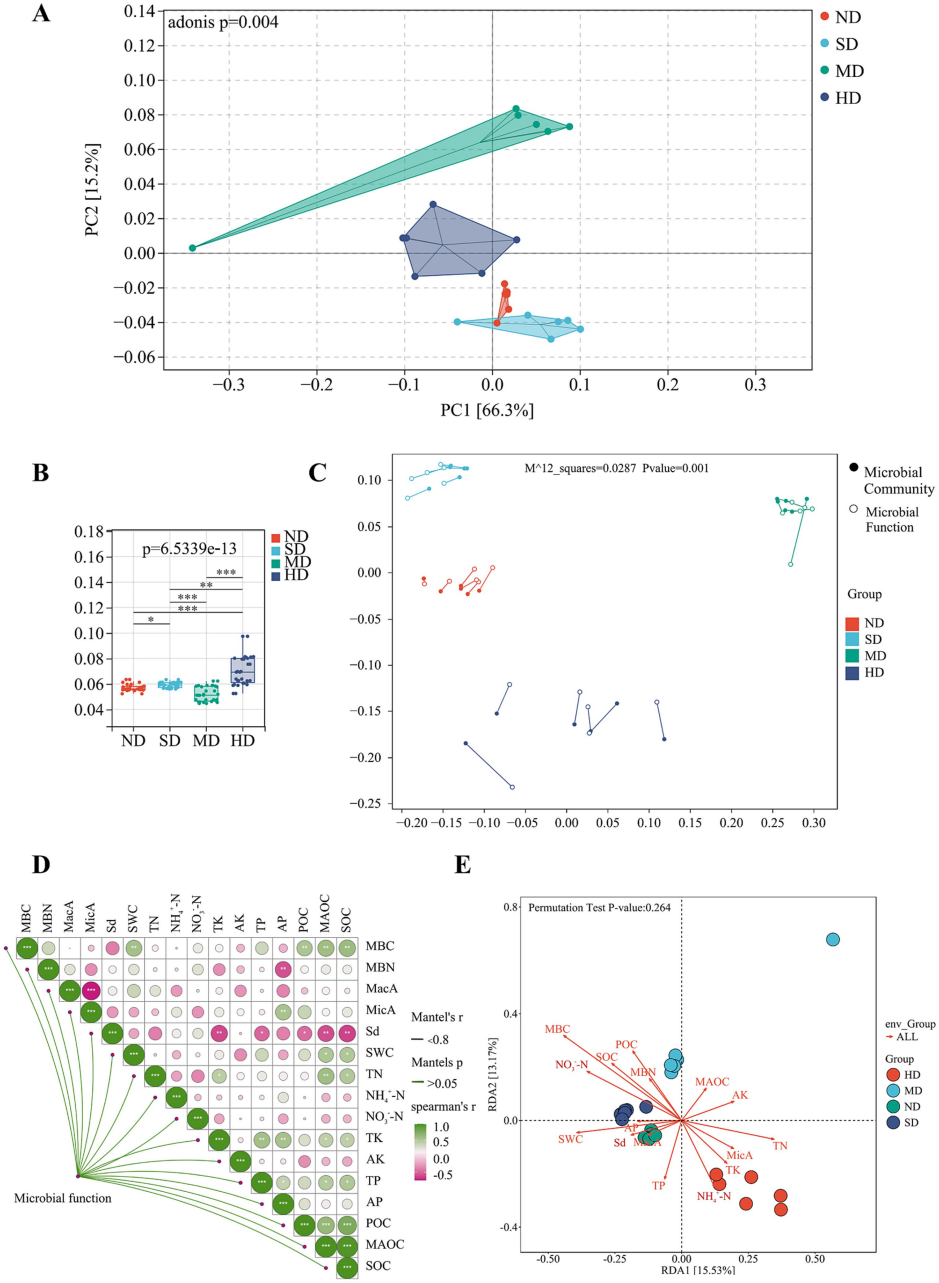
Microbial community structure and functional potential diagram. **(A)** shows beta diversity analysis (functional) ordination analysis, **(B)** shows beta diversity analysis (functional) distance difference analysis, **(C)** shows species-function correspondence analysis (Procrustes Micro analysis), **(D)** shows microbial functional gene-physicochemical joint analysis (Mantel Test analysis), and **(E)** shows microbial functional gene-physicochemical joint analysis (CCA_RDA analysis). MBC: microbial biomass carbon; MBN: microbial biomass nitrogen; MacA: macroaggregate; MicA: microaggregate; Sd: soil bulk density; SWC: soil water content; TN: total nitrogen; NH_4_^+^-N: ammonium nitrogen; NO_3_^−^-N: nitrate nitrogen; TK: total potassium; AK: available potassium; TP: total phosphorus; AP: available phosphorus; POC: particulate organic carbon; MAOC: mineral-associated organic carbon; SOC: soil organic carbon.

The *β*-diversity analysis, based on Bray-Curtis distance, revealed significant differences in microbial functional gene composition among grasslands with varying degradation levels (PERMANOVA, *p* = 0.001) (Figure 2B). Principal coordinate analysis (PCoA) demonstrated that samples exhibited a separation pattern along the first principal coordinate axis (PC1), which aligned with the degradation gradient, with PC1 accounting for 66.3% of the total variation (Figure 2A). Procrustes analysis indicated a significant correlation between microbial community structure and functional gene profiles (*M*^2^ = 0.0287, *p* = 0.001) (Figure 2C). Mantel test analysis revealed no significant correlations between the functional gene distance matrix and individual environmental variables (Figure 2D). Despite the absence of significant bivariate correlations in Mantel tests, redundancy analysis (RDA) identified key environmental factors associated with variations in functional profiles. In the RDA model, MBC exhibited the highest loading value (−0.83) on the primary ordination axis (RDA1), explaining an individual variation of 25.3% (*r*^2^ = 0.253, *p* = 0.055). All other soil physicochemical indicators demonstrated lower explanatory power in the model compared to MBC (Figure 2E).

### 3.3 Changes in abundance of functional genes related to carbon and nitrogen metabolism

**Figure 3:**
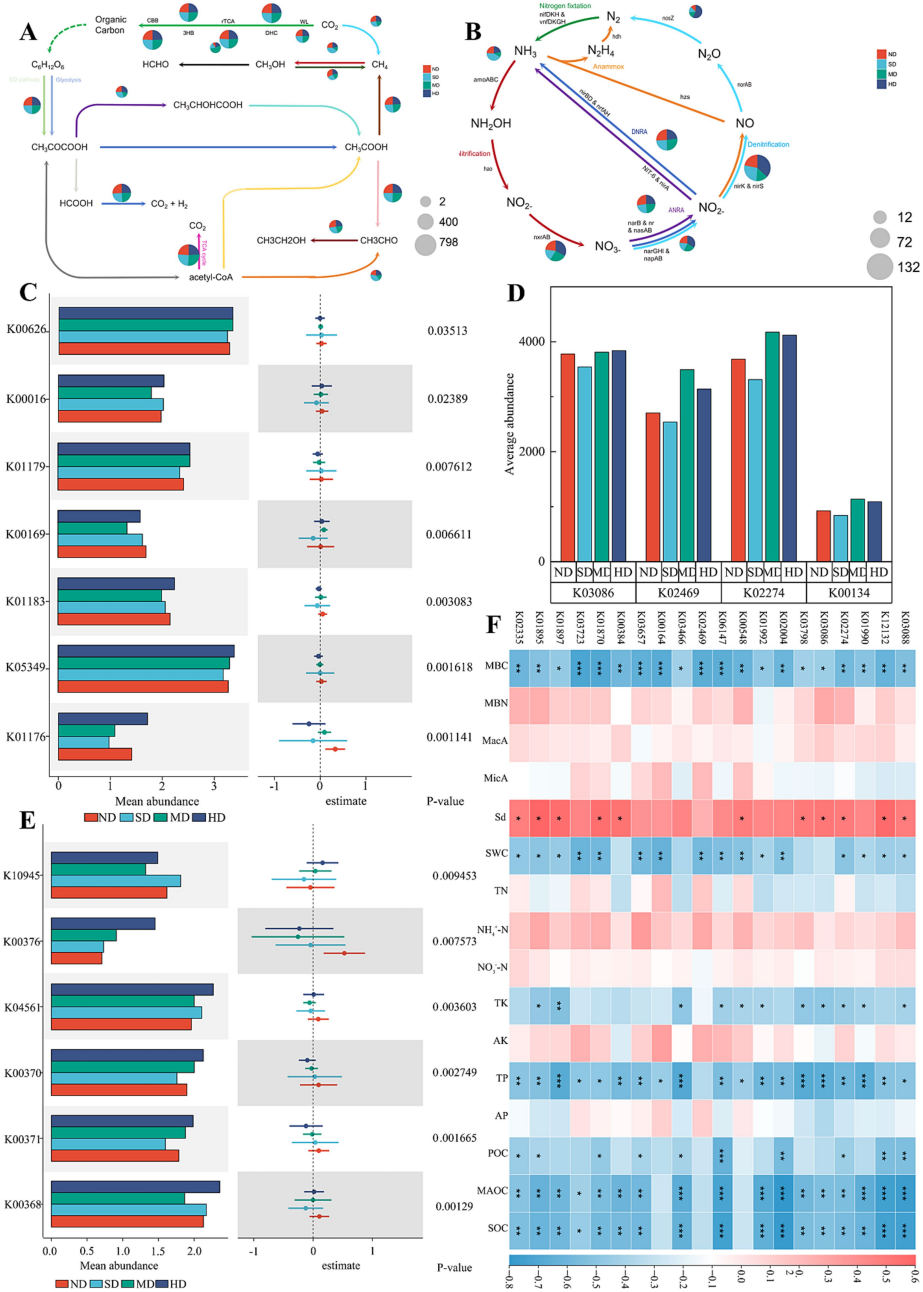
Correlation diagram of functional genes in carbon and nitrogen cycles with their functions and physicochemical factors. **(A)** shows the carbon cycle pathway diagram, **(B)** shows the nitrogen cycle pathway diagram, **(C)** displays the abundance of functional genes related to the carbon cycle, **(D)** presents the abundance of functional genes associated with microbial maintenance metabolism, **(E)** illustrates the abundance of functional genes related to the nitrogen cycle, and **(F)** shows the combined analysis (correlation analysis) of microbial functional genes and physicochemical properties.

The abundance of carbon metabolism-related functional genes exhibited significant variations along the grassland degradation gradient (Figure 3A). The porA gene (K00169), which encodes pyruvate-ferredoxin oxidoreductase, demonstrated the highest abundance at the ND stage, followed by a gradual decrease as degradation severity increased. In contrast, the ldh gene (K00016), encoding L-lactate dehydrogenase, and the atoB gene (K00626), encoding acetyl-CoA acetyltransferase, displayed significantly higher abundances at the MD and HD stages compared to the ND stage (Figure 3C). Additionally, the bglX gene (K05349), which encodes *β*-glucosidase, and the endoglucanase gene (K01179) were significantly enriched at the MD and HD stages. Notably, some basal metabolic-related genes, including the gapA gene (K00134) encoding glyceraldehyde-3-phosphate dehydrogenase, the rpoD gene (K03086) encoding RNA polymerase σ70 factor, the gyrA gene (K02469) encoding DNA replicase, and the coxA gene (K02274) encoding a key component of the aerobic respiratory chain, maintained high abundance across all degradation stages and exhibited significant negative correlations with MBC (Figures 3D,F).

The response of nitrogen cycle functional genes exhibited distinct patterns (Figure 3B). In the denitrification pathway, the abundances of genes encoding nitrate reductase (narG, K00370 and narH, K00371), nitrite reductase (nirK, K00368), nitric oxide reductase (norB, K04561), and nitrous oxide reductase (nosZ, K00376) peaked during the HD stage (Figure 3E). In contrast, the abundance of the gene encoding ammonia monooxygenase (amoA, K10945) significantly decreased after the SD stage (Figure 3E). Functional genes involved in carbon and nitrogen metabolism displayed differentiated variation patterns along the degradation gradient. Key carbon metabolism genes (such as porA, ldh, and atoB) began to show significant changes as early as the MD stage, while the abundance of denitrification genes (including narG, nirK, norB, and nosZ) primarily increased during the HD stage. Notably, the decline in amoA gene abundance commenced at the SD stage, preceding the increase in denitrification gene abundance.

### 3.4 SEM of the association between microbial functional modules and soil carbon-nitrogen

**Figure 4:**
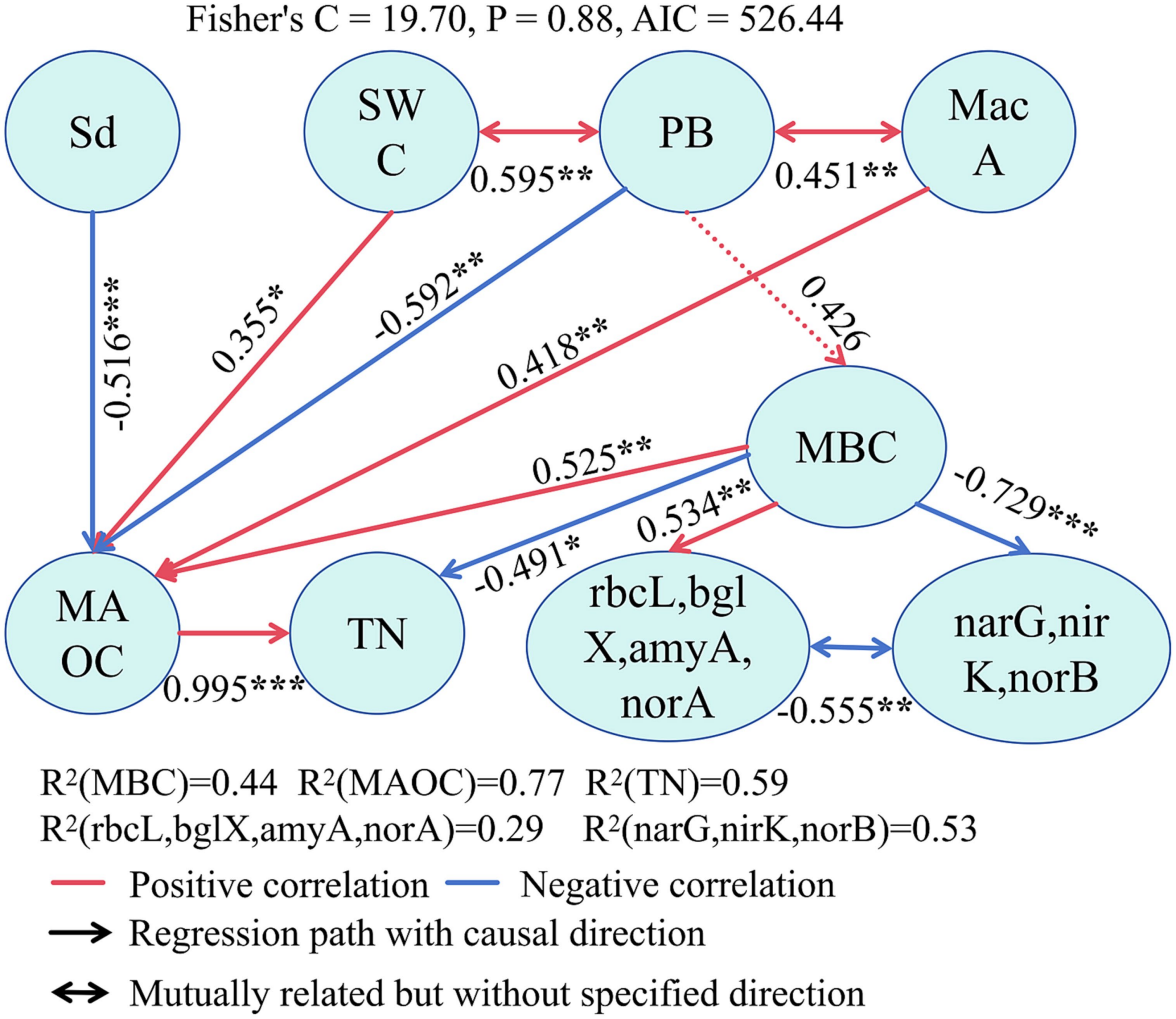
SEM segmented structural equation model diagram. Only paths with *p* < 0.1 are shown; other paths are presented in Supplementary Table 3. * indicates *p* < 0.05, ** indicates *p* < 0.01, *** indicates *p* < 0.001. The carbon-energy regulation module consists of rbcL + bglX + amyA + norA, while the denitrification functional module comprises narG + nirK + norB.

The SEM revealed a hierarchical pattern of conditional associations (Figure 4). In the plant–soil–microbe layer, PB was positively correlated with SWC (*r* = 0.595, *p <* 0.01) and MacA (*r* = 0.451, *p <* 0.05), whereas no significant covariation was observed with Sd. MBC showed a marginal positive association with PB (*β* = 0.426, *p <* 0.1), while the direct associations of SWC, MacA, and Sd with MBC did not achieve statistical significance.

In the microbial–functional layer, MBC was positively associated with the carbon-energy regulation module (rbcL, bglX, amyA, norA) (*β* = 0.525, *p <* 0.01), and negatively associated with the denitrification functional module (narG, nirK, norB) (*β* = −0.729, *p <* 0.001). A negative covariance was observed between these two functional modules (standardized covariance = −0.555, *p <* 0.01).

In the carbon pool layer, several variables showed conditional associations with MAOC. MBC (*β* = 0.525, *p <* 0.01), MacA (*β* = 0.418, *p <* 0.01), and SWC (*β* = 0.355, *p <* 0.05) were all positively associated with MAOC, whereas Sd was negatively associated with MAOC (*β* = −0.516, *p <* 0.001). PB showed a negative conditional association with MAOC (*β* = −0.592, *p <* 0.01); however, the bivariate correlation between PB and MAOC was not significant (Supplementary Table 6). Neither the carbon-energy regulation module nor the denitrification functional module showed a significant direct association with MAOC.

In the nitrogen pool layer, MAOC showed a positive conditional association with TN (*β* = 0.995, *p <* 0.001); the bivariate correlation between MAOC and TN was *r* = 0.599 (Supplementary Table 6). Given the limited sample size, this conditional coefficient may be inflated and should be interpreted as a directional reference. MBC showed an independent negative association with TN (*β* = −0.491, *p <* 0.05), while the bivariate correlation between MBC and TN was not significant (Supplementary Table 6). The direct paths from Sd and SWC to TN were not significant. Across all regression equations, VIF values remained below 2.35 (Supplementary Table 5), and the d-separation test indicated no significant omitted paths (all *p >* 0.1; Supplementary Table 4). Univariate regression results and the full correlation matrix are presented in Supplementary Tables 7 and 8.

## 4 Discussion

### 4.1 Correlation between grassland degradation level and soil carbon-nitrogen content

To understand the changes in soil carbon and nitrogen pools during grassland degradation, it is essential to focus on their nonlinear characteristics along the degradation gradient. Most previous studies have reported a continuous decline in soil organic carbon due to grassland degradation (Zhang et al., 2020; Shi et al., 2025). However, they often only compared the endpoints of degraded and non-degraded states, overlooking potential turning points during the degradation process. The gradient design of this study reveals a non-monotonic trajectory of MAOC, which first increases and then decreases. This phenomenon can be explained by phased changes in three aspects: plant carbon input, aggregate protection, and microbial residue accumulation (see Supplementary Tables 1 and 2). In the MD stage, although PB has decreased to about 30% of the ND stage, it can still maintain a certain level of carbon supply. At this stage, the fragmentation of MacA and the increase in small aggregates may expose new mineral binding sites, while the accumulation of microbial residues could temporarily enhance carbon storage (Jia et al., 2025). The combined effect of these factors may represent the underlying mechanism for the temporary accumulation of MAOC. By the HD stage, PB further decreases to approximately 20% of the ND stage level, MBC sharply declines, and MAOC subsequently collapses. Yu et al. (2025) also observed a significant decrease in microbial residue carbon in grassland extreme drought experiments, indicating that extreme stress can lead to a net loss of stable carbon pools. This nonlinear trajectory resembles observations of temporary soil carbon increases in certain forest or farmland ecosystems during the initial disturbance phase (Hu et al., 2024; Wang et al., 2024), suggesting that the balance between carbon sequestration and loss undergoes phased shifts during the degradation process.

In terms of carbon-nitrogen coupling, the fate of nitrogen released from MAOC decomposition is a crucial factor in evaluating the nitrogen pool dynamics of degraded grasslands. During the HD stage, MAOC exhibited a significant decline; however, TN did not decrease synchronously to levels below those observed in the ND stage. This decoupling phenomenon suggests that organic nitrogen released from MAOC decomposition is not necessarily lost from the system in gaseous forms. Inorganic nitrogen data indicate that soil ammonium (NH₄^+^-N) content during the HD stage was slightly higher than in other stages, while nitrate (NO_3_^−^-N) levels showed no significant differences across stages (Supplementary Table 2). Although the intergroup differences did not reach statistical significance, the substantial decline in MAOC during the HD phase was not accompanied by a corresponding reduction in TN. This pattern suggests that most of the released organic nitrogen may have been retained in the soil in inorganic forms, indicating a transformation from stable organic pools to reactive inorganic pools rather than a net nitrogen loss from the ecosystem. While the possibility of partial nitrogen loss through denitrification in gaseous form cannot be entirely dismissed (as denitrification gene abundance increased during the HD phase), the existing data more strongly support “form transformation” as the primary process. This understanding challenges the oversimplified view that “MAOC disintegration necessarily leads to a net reduction in soil nitrogen pools” and has significant implications for nitrogen management in degraded grasslands.

SEM further revealed distinct associational patterns for carbon and nitrogen pool dynamics. The variation in MAOC was jointly associated with MBC, MacA, Sd, and SWC, with Sd exhibiting the most stable negative association. In terms of TN response, these physical factors primarily influenced the nitrogen pool through the mediation of MAOC. This pattern aligns with the theoretical expectation that mineral-associated organic matter serves as the main carrier of soil nitrogen. MAOC is predominantly formed through the binding of nitrogen-rich microbial residues to mineral surfaces, typically exhibiting a lower carbon-to-nitrogen ratio than particulate organic matter. While MBC showed a positive association with MAOC, it demonstrated an independent negative association with TN. This dual pattern suggests that MBC decline may be linked to carbon and nitrogen pools through two relatively independent pathways: on one hand, by weakening the replenishment of microbial residues to MAOC, and on the other hand, by coupling with enhanced denitrification potential, which may directly contribute to gaseous nitrogen loss or transformation.

PB exhibited a continuous decline along the degradation gradient, whereas MAOC demonstrated a nonlinear pattern characterized by an initial increase followed by a decrease. Notably, there was no significant simple linear correlation between the two variables. This observation suggests that the dynamics of MAOC are not directly driven by PB in a linear manner; instead, they are regulated by MBC-mediated nonlinear processes. During the MD stage, despite a significant reduction in PB, MBC remained relatively stable, resulting in the accumulation of MAOC. Conversely, in the HD stage, as PB dropped to extremely low levels, a threshold-like decline in MBC coincided with increased soil compaction (evidenced by elevated Sd and decreased MacA), ultimately triggering the collapse of MAOC. Thus, the abrupt decline of MBC may represent a critical transition point associated with the shift from MAOC accumulation to loss, while the continuous decline of PB represents a prerequisite rather than a direct factor in this process. In the SEM analysis, the relationship between PB and MAOC reveals a noteworthy statistical phenomenon: after controlling for variables such as MBC, PB exhibits a significant negative partial correlation with MAOC, while their simple correlation remains non-significant. The emergence of this suppression effect further underscores the importance of MBC as a mediating variable; the decline of PB itself was not directly associated with the loss of MAOC; rather, MAOC decline was observed only when MBC also showed threshold-like attenuation. None of the direct pathways from physical drivers to MBC reached significance in the SEM, which indirectly indicates that the decline of MBC is primarily associated with nonlinear processes along the degradation gradient, rather than linearly linked to individual physical factors. Notably, SWC and MBC displayed a significant positive correlation in simple correlation, but their direct effect became non-significant after controlling for PB, suggesting that the influence of SWC on MBC may be primarily mediated through PB. The negative association between Sd and MAOC remained significant after controlling for MBC, suggesting that Sd is associated with MAOC independently of the MBC-mediated pathway, potentially through direct disruption of mineral-organic binding sites or altered pore structures.

In summary, this study demonstrates that MAOC emerged as the core coupling variable linking carbon and nitrogen dynamics along the degradation gradient. The increase in Sd and the decay of MBC are significantly correlated with the decline of MAOC, which in turn shows a positive association with TN reduction. However, TN during the HD phase only reverted to ND levels rather than further decreasing, suggesting that MAOC disintegration does not necessarily lead to net nitrogen loss. The fate of organic nitrogen released during disintegration ultimately determines the final nitrogen pool outcome. The inorganic nitrogen data in this study supports “form transformation” as the primary pathway. When assessing carbon and nitrogen pools in degraded grasslands, monitoring MAOC can provide synchronous references for evaluating the stability changes of both carbon and nitrogen.

### 4.2 The shift of microbial functional assembly from stochastic to deterministic

Understanding how the assembly mechanisms of microbial functional communities shift along environmental stress gradients is a central question in community assembly research. This study demonstrates that, along the grassland degradation gradient, the assembly processes of microbial functional gene communities transition from stochastic-dominated to deterministic-dominated mechanisms. This finding supports the predictions outlined in Hypothesis (1). Such transitions from stochastic to deterministic assembly have been widely documented in other studies examining environmental gradients, including elevation gradients (Yao et al., 2025), soil depth gradients (Jiao et al., 2018), and agricultural management gradients (Liu W. et al., 2021). As environmental stress intensifies, deterministic processes frequently dominate community assembly. The contribution of this study lies in validating this pattern along a grassland degradation gradient at the microbial functional gene level and quantitatively identifying the specific transitional phase—the MD phase—when deterministic filtering reaches its peak intensity.

It is worth in-depth consideration that the transition in assembly processes does not follow a monotonic intensification but rather exhibits nonlinear characteristics. During the HD stage, SES values did not persistently deviate from null model expectations; instead, they returned to non-significant levels. This pattern suggests that community assembly rules may undergo a secondary shift during extreme degradation stages. We propose two possible explanations for this phenomenon: (1) Under extreme degradation, functional gene sets tend to become simplified, with increased community variability masking deterministic signals by stochastic noise, while actual environmental filtering effects may remain strong; (2) The community has transitioned beyond the strong filtering window of the MD stage into a functionally stable but inefficient “pseudo-steady state,” where the intensity of environmental filtering has indeed weakened. Due to the cross-sectional design of this study, it is currently impossible to distinguish between these two mechanisms. If the former holds true, the community assembly process in the HD stage may still be strongly driven by deterministic factors, though undetected by the current statistical framework. Conversely, if the latter is the case, it implies that the community has transitioned into a new state after overcoming the strong filtering of the MD stage, with both its assembly mechanisms and functional consequences differing from previous transitional phases. Subsequent time-series analyses or experimental manipulation studies will be crucial for confirming the ecological nature of this nonlinear pattern.

The shift in functional assembly mechanisms exhibits a noteworthy synchrony with the nonlinear dynamics of MAOC at critical nodes. The MD phase represents both the stage with the strongest deterministic filtering and the peak accumulation phase of MAOC. This temporal correlation suggests that intensified environmental filtering may be associated with microbially-mediated carbon transformation processes through the selection of specific functional taxa, thereby establishing a link with MAOC accumulation. Similarly, Tian et al. (2021) observed in mountain soils that community assembly shifts driven by pH and organic carbon attributes were closely associated with variations in carbon decomposition functionality. However, this study only reports the existence of this association; whether deterministic filtering is directly linked to MAOC accumulation—rather than merely coinciding with it—remains to be experimentally verified.

In summary, this study demonstrates that along the gradient of grassland degradation, the shift in microbial functional assembly from stochastic to deterministic processes does not exhibit a monotonic strengthening pattern. Notably, the MD stage shows the strongest deterministic signal, whereas the HD stage returns to non-significant levels. The MD stage may represent a critical window for functional community reorganization. However, this transition pattern, along with its relationship to MAOC dynamics, necessitates validation through longitudinal data to differentiate between possibilities such as pseudo-steady-state masking, statistical artifacts, and genuine state transitions. These findings provide a framework for understanding the differential responses of microbial communities in heterogeneous degradation patches and establish a foundation for subsequent analyses of variations in carbon and nitrogen metabolic genes.

### 4.3 Shifts in carbon-nitrogen metabolic gene profiles and their negative covariance with MBC

The systematic variation of carbon and nitrogen metabolic gene profiles along the degradation gradient reflects the metabolic strategy shift of microbial communities from carbon sufficiency to energy limitation. The consistently downregulated aerobic oxidation gene (porA) and the significantly upregulated fermentation genes (ldh, atoB) observed in this study collectively highlight the core dilemma faced by microorganisms: as MBC continuously declines, energy acquisition pathways are compelled to transition from efficient aerobic respiration to less efficient fermentation in order to sustain basic maintenance metabolism. The SEM revealed a significant negative covariance between the carbon-energy regulation module and the denitrification functional module, with MBC emerging as the central variable associated with this functional divergence. MBC was positively associated with the carbon-energy regulation module and negatively associated with the denitrification module. Neither functional module showed a direct association with MAOC or TN, thereby suggesting the hypothesis that functional gene restructuring is a concomitant response to MBC decline, rather than an independently driven process.

From the perspective of biochemical coupling, fermentation products are generally regarded as preferred electron donors and carbon skeletons for denitrifying microorganisms (Li et al., 2023; Luo et al., 2023). In this study, the HD phase demonstrated both an increased abundance of fermentation genes and the highest abundance of denitrification genes, with a negative covariance observed between them. This pattern aligns directionally with the theoretical expectations of the fermentation-denitrification coupling mechanism; however, the underlying logic may not merely involve a substrate supply relationship. When the MBC declines to a critical level, microorganisms, constrained by energy limitations, increasingly depend on less efficient fermentation pathways. The metabolic intermediates produced could theoretically supply substrates for denitrifying microbial communities. Nonetheless, this coordinated shift in metabolic potential more likely reflects the overall deterioration of the community’s energy status rather than a direct facilitation of one functional group by another. Similar phenomena have been observed in degraded grasslands (Lei et al., 2021) and saline soils (Ning et al., 2023), although these studies primarily reported correlations at the gene abundance level, lacking direct validation through flux data. Therefore, whether fermentation-denitrification coupling genuinely occurs in this system still requires evidence at the metabolic flux level.

A central finding of this study is that, although the abundances of both the carbon-energy regulation module and the denitrification functional module showed significant variations along the degradation gradient and were strongly correlated with MBC, neither showed a direct association with MAOC or TN in the SEM. This pattern suggests that the restructuring of functional gene profiles does not directly translate into changes in stable carbon and nitrogen pools; rather, it is indirectly linked to these pools through MBC as a shared hub. The threshold-like decline of MBC was closely associated with reductions in both MAOC and TN, while the observed shifts in functional genes—such as weakened aerobic oxidation, enhanced fermentation, and elevated denitrification genes—may represent accompanying signals of microbial metabolic strategy adjustments during MBC depletion. Although these signals cannot independently explain the loss of carbon and nitrogen pools, their changes precede the collapse of stable carbon pools, making them potential indicators for microbial energy stress and system functional degradation. This interpretation adjusts the initial expectation based on gene abundance; the value of gene responses lies in their potential indicator function rather than in directly driving functionality.

In summary, this study reveals, at the functional gene level, the parallel changes of weakened aerobic oxidation and enhanced fermentation in carbon metabolism, alongside increased denitrification gene abundance. Additionally, it highlights the negative covariance between these processes, linked through MBC. However, it is important to note that gene abundance reflects metabolic potential rather than actual flux, with multiple intermediate steps existing in the transformation from genetic potential to material stocks. Future research should integrate metatranscriptomics and stable isotope probing techniques to verify the relationships between functional gene expression and actual carbon-nitrogen fluxes, thereby advancing statistical covariation patterns toward a mechanistic understanding.

### 4.4 Implications for grassland monitoring and research limitations

#### 4.4.1 Potential microbial monitoring indicators

Traditional assessments of grassland degradation primarily rely on static indicators, such as vegetation coverage and aboveground biomass. A fundamental limitation of these metrics is that they reflect the outcomes of ecosystem decline rather than its precursors. This study reveals that MBC consistently declines along the degradation gradient, with a sharp attenuation occurring at the HD stage. Notably, the rate of change in MBC precedes the collapse of MAOC. Concurrently, the abundance ratio of fermentation genes to aerobic oxidation genes begins to increase as early as the MD stage, while the ratio of denitrification genes to nitrification genes rises significantly during the HD stage. By integrating these dynamics, we propose that the sustained decline rate of MBC and the shifts in these functional gene ratios may serve as potential indicators of declining soil carbon-nitrogen stability in grasslands, as they temporally precede substantial losses in stable carbon pools.

The distinctive feature of this indicator system lies in its integration of MBC—a central functional variable—with gene ratios that reflect shifts in metabolic strategies, while simultaneously monitoring changes in carbon and nitrogen stability through MAOC observations. SEM further elucidates that MBC serves as the pivotal node linking functional differentiation with the attenuation of carbon and nitrogen pools, whereas Sd emerges as the most sensitive physical indicator for MAOC loss. It is important to emphasize that the aforementioned functional gene ratios reflect metabolic potential at the DNA level, and their specific thresholds as potential indicators require validation through independent datasets. Furthermore, while soil microbial indicators are receiving increasing attention in health assessments (Jia et al., 2025), the incorporation of gene ratios into field rapid testing still necessitates the development of cost-effective and standardized detection protocols.

#### 4.4.2 Research limitations

This study has the following limitations: (1) The analysis concentrated on the genetic potential of microbial functional genes, rather than on actual metabolic activity or material fluxes. Changes in gene abundance merely reflect alterations in metabolic potential and do not necessarily signify corresponding changes in process rates. (2) The SEM utilized a space-for-time substitution design, wherein the path coefficients indicate the conditional covariation among variables, but do not directly verify causal directions. (3) The increase in MAOC during the MD stage was not directly measured to determine whether it resulted from greater microbial necromass accumulation, such as amino sugars, or from enhanced mineral protection. Similarly, the decline in MAOC during the HD stage, which may involve microbial “mining” of pre-existing MAOC, necessitates experimental verification. (4) This study employed a space-for-time substitution design, which cannot adequately capture the temporal dynamics of the same community during degradation. Different degradation stages may represent patches with distinct historical trajectories rather than a continuous evolution of the same system. Therefore, the observed shifts in assembly mechanisms and nonlinear changes in MAOC should be interpreted as correlational patterns, the temporal manifestations of which require confirmation through longitudinal studies. (5) At the HD stage, the levels of MAOC had significantly decreased below those of ND, while TN returned only to its initial levels. The inorganic nitrogen data in this study directionally support the notion that most of the released organic nitrogen was retained in inorganic forms; however, inter-group differences were not significant, and the proportion of gaseous losses remains unquantified. Resolving this issue is crucial for assessing the fate of nitrogen pools in degraded grasslands, which necessitates future elucidation through combined measurements of denitrification fluxes and nitrogen stable isotope tracing. (6) In this study, the conditional path coefficient between TN and MAOC was anomalously close to 1.0, which is significantly higher than their zero-order correlation coefficient. Considering the small sample size (n = 24) and the potential for complex collinearity among variables, this coefficient is likely overestimated. Therefore, we regard the conditional MAOC-TN association solely as a directional reference, and its specific numerical value should not be adopted. This relationship necessitates validation in larger samples or multiple independent datasets in future research. (7) This study found that the carbon-energy regulation module and the denitrification module exhibited no direct significant pathways to MAOC and TN. However, this does not preclude the possibility that variations in functional gene abundance may predict the dynamics of carbon and nitrogen pools over longer time scales or under different environmental conditions. Our conclusions are constrained to the temporal and spatial scales of this study. Specifically, within the degradation gradient of typical Inner Mongolia grasslands, MBC was more closely associated with changes in carbon and nitrogen pools compared to functional gene abundance. Furthermore, changes in functional genes should be viewed as responsive signals accompanying the attenuation of MBC.

Future research should integrate metatranscriptomics, stable isotope probing, and multi-site flux observations to verify causal relationships and establish robust indicator systems. Long-term *in situ* experiments or restoration tracking studies will help distinguish the temporal sequence of degradation stages from system thresholds, thereby providing a more solid scientific foundation for precise diagnosis and zonal management of grassland degradation.

## 5 Conclusion

This study revealed that: (1) The assembly process of microbial functional gene communities transitioned from being predominantly stochastic to deterministic, with the most pronounced deterministic filtering effect observed during the MD stage. (2) MBC was a central variable linking microbial functional differentiation to changes in soil carbon and nitrogen pools. The threshold-like decline of MBC was closely associated with the collapse of stable carbon and nitrogen pools. (3) MAOC exhibited a nonlinear pattern along the degradation gradient, initially increasing before decreasing, while TN demonstrated a synchronous increase during the MD stage before declining to ND levels in the HD stage. Nitrogen released from MAOC decomposition primarily remained in the soil in inorganic forms. (4) The continuous decline of MBC and changes in functional gene ratios may serve as potential indicators of declining carbon and nitrogen stability in grassland soils.

## Data Availability

The datasets presented in this study can be found in online repositories. The names of the repository/repositories and accession number(s) can be found in the article/Supplementary material.
