# Supplementary material for: Microbial functional gene assembly is associated with soil carbon and nitrogen dynamics during grassland degradation
Source: Front Microbiol. 2026 Jul 8;17:1878594. doi: 10.3389/fmicb.2026.1878594 (PMC13388392; doi:10.3389/fmicb.2026.1878594)
Supplement: Supplementary file 1 [file Table_1.DOCX]

Supplementary Material

Table 1 Plant Data Sheet

| Treatment | Biomass(g/m^2^) |
| --- | --- |
| ND | 123.32±43.17a |
| SD | 67.97±36.05b |
| MD | 37.79±5.77bc |
| HD | 24.68±9.18c |

Note: ND: Non-degraded; SD: Slightly degraded; MD: Moderately degraded; HD: Heavily degraded, the same below. Different lowercase letters in the same column indicate no significant difference (P≤0.05)

Table 2 Soil Physicochemical Properties

| Treatment | MBC(mg/kg) | MBN(mg/kg) | MacA % | MicA % | Sd g/cm^3^ | SWC % | TN(g/kg) | NH_4_^+^-N(g/kg) | NO_3_^-^-N(g/kg) |
| --- | --- | --- | --- | --- | --- | --- | --- | --- | --- |
| ND | 131.51±8.04a | 22.34±0.72a | 41.91±2.94a | 39.19±2.27b | 1.45±0.03a | 9.72±1.07a | 2.36±0.14b | 181.13±15.58a | 272.91±10.21a |
| SD | 127.37±6.23a | 10.37±1.42c | 34.58±1.56b | 51.55±2.45a | 1.36±0.05a | 8.34±0.82a | 2.35±0.12b | 197.02±24.94a | 248.79±9.51a |
| MD | 118.35±10.53a | 17.36±1.47b | 36.85±1.36ab | 50.08±2.72a | 1.38±0.04a | 7.36±0.91ab | 2.9±0.21a | 191.45±24.63a | 272.13±27.93a |
| HD | 63.3±9.39b | 10.11±0.66c | 35.19±1.58b | 47.97±2.77a | 1.47±0.04a | 5.61±0.43b | 2.39±0.19b | 217.61±23.31a | 267.49±20.12a |

Continued Table 2 Soil Physicochemical Properties

| Treatment | TK(g/kg) | AK(mg/kg) | TP(g/kg) | AP(mg/kg) | POC(g/kg) | MAOC(g/kg) | SOC(g/kg) | POC% | MAOC% |
| --- | --- | --- | --- | --- | --- | --- | --- | --- | --- |
| ND | 6.08±0.24a | 2.31±0.21a | 0.25±0.01a | 5.01±0.91b | 2.43±0.23b | 10.32±0.75b | 12.76±0.86b | 19.17±1.29a | 80.83±1.29a |
| SD | 6.7±0.23a | 2.31±0.24a | 0.26±0.01a | 16.22±2.67a | 3.53±0.19a | 12.45±1.35ab | 15.98±1.34ab | 22.86±2.17a | 77.14±2.17a |
| MD | 7.01±0.47a | 2.98±0.22a | 0.27±0.02a | 13.06±1.16a | 3.01±0.3ba | 14.08±1.37a | 17.09±1.43a | 17.94±1.75a | 82.06±1.75a |
| HD | 6.47±0.56a | 2.82±0.21a | 0.23±0.01a | 12.17±1.54a | 1.35±0.08c | 7.05±0.84c | 8.4±0.82c | 17.04±2.24a | 82.96±2.24a |

Note: Different lowercase letters in the same column indicate no significant difference (*P* < 0.05). MBC: microbial biomass carbon; MBN: microbial biomass nitrogen; MacA: macroaggregate; MicA: microaggregate; Sd: soil bulk density; SWC: soil water content; TN: total nitrogen; NH_4_^+^-N: ammonium nitrogen; NO_3_^-^-N: nitrate nitrogen; TK: total potassium; AK: available potassium; TP: total phosphorus; AP: available phosphorus; POC: particulate organic carbon; MAOC: mineral-associated organic carbon; SOC: soil organic carbon. The same below.

Table 3 SEM Path Coefficients

| Response | Predictor | Unstd_Estimate | Std_Error | Std_Estimate | P_value | Significance |
| --- | --- | --- | --- | --- | --- | --- |
| MBC | PB | 0.3112 | 0.1687 | 0.426 | 0.0807 | . |
| MBC | SWC | 4.1391 | 3.0316 | 0.296 | 0.1881 | ns |
| MBC | MacA | -1.6186 | 1.2925 | -0.253 | 0.2257 | ns |
| MBC | Sd | -73.6362 | 61.9962 | -0.217 | 0.2496 | ns |
| C_module | MBC | 0.0261 | 0.0088 | 0.534 | 0.0071 | ** |
| N_module | MBC | -0.0316 | 0.0063 | -0.729 | 1e-04 | *** |
| MAOC | MBC | 0.0565 | 0.0162 | 0.525 | 0.0026 | ** |
| MAOC | MacA | 0.2887 | 0.095 | 0.418 | 0.007 | ** |
| MAOC | Sd | -18.8862 | 4.538 | -0.516 | 6e-04 | *** |
| MAOC | SWC | 0.5352 | 0.2244 | 0.355 | 0.0282 | * |
| MAOC | PB | -0.0465 | 0.0129 | -0.592 | 0.0021 | ** |
| TN | MBC | -0.0064 | 0.0025 | -0.491 | 0.0181 | * |
| TN | Sd | 0.2565 | 0.85 | 0.058 | 0.7661 | ns |
| TN | SWC | -0.0337 | 0.0328 | -0.183 | 0.318 | ns |
| TN | MAOC | 0.1212 | 0.0273 | 0.995 | 3e-04 | *** |
| ~~PB | ~~SWC | 0.5952 | NA | 0.595 | 0.0022 | ** |
| ~~PB | ~~MacA | 0.4506 | NA | 0.451 | 0.0271 | * |
| ~~PB | ~~Sd | -0.0043 | NA | -0.004 | 0.9839 | ns |
| ~~SWC | ~~MacA | 0.2606 | NA | 0.261 | 0.2187 | ns |
| ~~SWC | ~~Sd | -0.1302 | NA | -0.13 | 0.5442 | ns |
| ~~MacA | ~~Sd | 0.2665 | NA | 0.266 | 0.2081 | ns |
| ~~C_module | ~~N_module | -0.5554 | NA | -0.555 | 0.003 | ** |

Note: C_module represents rbcL+bglX+amyA+norA, and N_module represents narG+nirK+norB. The same applies to the following tables.

Table 4 d-separation test table

| Independ.Claim | Test.Type | DF | Crit.Value | P.Value |
| --- | --- | --- | --- | --- |
| TN ~ PB + ... | coef | 18.0000 | 0.4021 | 0.6923 |
| C_module ~ PB + ... | coef | 21.0000 | -0.2090 | 0.8365 |
| N_module ~ PB + ... | coef | 21.0000 | -0.3141 | 0.7565 |
| C_module ~ SWC + ... | coef | 21.0000 | 0.9878 | 0.3345 |
| N_module ~ SWC + ... | coef | 21.0000 | -1.0307 | 0.3144 |
| TN ~ MacA + ... | coef | 18.0000 | 1.1497 | 0.2653 |
| C_module ~ MacA + ... | coef | 21.0000 | 0.2571 | 0.7996 |
| N_module ~ MacA + ... | coef | 21.0000 | -1.4697 | 0.1565 |
| C_module ~ Sd + ... | coef | 21.0000 | -1.0825 | 0.2913 |
| N_module ~ Sd + ... | coef | 21.0000 | 0.3093 | 0.7602 |
| C_module ~ MAOC + ... | coef | 17.0000 | 0.2530 | 0.8033 |
| N_module ~ MAOC + ... | coef | 17.0000 | 0.1687 | 0.8680 |
| C_module ~ TN + ... | coef | 18.0000 | -1.1616 | 0.2606 |
| N_module ~ TN + ... | coef | 18.0000 | -0.1845 | 0.8557 |

Table 5 VIF Test Table

| Regression | Predictor | VIF |
| --- | --- | --- |
| MBC | PB | 1.81 |
| MBC | SWC | 1.59 |
| MBC | MacA | 1.38 |
| MBC | Sd | 1.13 |
| MAOC | MBC | 1.79 |
| MAOC | MacA | 1.5 |
| MAOC | Sd | 1.21 |
| MAOC | SWC | 1.75 |
| MAOC | PB | 2.14 |
| TN | MBC | 1.68 |
| TN | Sd | 1.7 |
| TN | SWC | 1.49 |
| TN | MAOC | 2.35 |

Table 6 Bivariate Correlations

| Var1 | Var2 | r | P_value | Significance |
| --- | --- | --- | --- | --- |
| PB | SWC | 0.595 | 0.0022 | ** |
| PB | MacA | 0.451 | 0.0271 | * |
| PB | Sd | -0.004 | 0.9839 | ns |
| SWC | MacA | 0.261 | 0.2187 | ns |
| SWC | Sd | -0.13 | 0.5442 | ns |
| MacA | Sd | 0.267 | 0.2081 | ns |
| PB | MBC | 0.489 | 0.0152 | * |
| SWC | MBC | 0.512 | 0.0106 | * |
| MacA | MBC | -0.041 | 0.8485 | ns |
| Sd | MBC | -0.324 | 0.1221 | ns |
| MBC | C_module | 0.534 | 0.0071 | ** |
| MBC | N_module | -0.729 | 1.00E-04 | *** |
| C_module | N_module | -0.711 | 1.00E-04 | *** |
| MBC | MAOC | 0.567 | 0.0038 | ** |
| MacA | MAOC | 0.085 | 0.6924 | ns |
| Sd | MAOC | -0.619 | 0.0013 | ** |
| SWC | MAOC | 0.448 | 0.0283 | * |
| PB | MAOC | 0.067 | 0.7549 | ns |
| MBC | TN | -0.039 | 0.8551 | ns |
| Sd | TN | -0.375 | 0.0713 | . |
| SWC | TN | 0.003 | 0.9878 | ns |
| MAOC | TN | 0.599 | 0.002 | ** |

Table 7 univariate_regressions table

| Response | Predictor | Intercept | Slope | R_squared | P_value | Significance |
| --- | --- | --- | --- | --- | --- | --- |
| MAOC | MBC | 4.2513 | 0.0611 | 0.322 | 0.0038 | ** |
| MAOC | PB | 10.6409 | 0.0053 | 0.005 | 0.7549 | ns |
| MAOC | Sd | 42.9169 | -22.6331 | 0.383 | 0.0013 | ** |
| MAOC | SWC | 5.7391 | 0.6749 | 0.2 | 0.0283 | * |
| MAOC | MacA | 8.795 | 0.0587 | 0.007 | 0.6924 | ns |
| TN | MAOC | 1.6996 | 0.0729 | 0.358 | 0.002 | ** |
| TN | MBC | 2.5571 | -5.00E-04 | 0.002 | 0.8551 | ns |
| TN | Sd | 4.8579 | -1.6706 | 0.14 | 0.0713 | . |
| TN | SWC | 2.4956 | 6.00E-04 | 0 | 0.9878 | ns |
| MBC | PB | 87.475 | 0.3572 | 0.239 | 0.0152 | * |
| MBC | SWC | 54.5288 | 7.1663 | 0.262 | 0.0106 | * |
| MBC | Sd | 265.7002 | -110.2331 | 0.105 | 0.1221 | ns |
| MBC | MacA | 119.933 | -0.2639 | 0.002 | 0.8485 | ns |

Table 8 Correlation Matrix

| Response | Predictor | Unstd_Estimate | Std_Error | P_value | Significance |
| --- | --- | --- | --- | --- | --- |
| MBC | (Intercept) | 222.295 | 90.0126 | 0.0232 | * |
| MBC | PB | 0.3112 | 0.1687 | 0.0807 | . |
| MBC | SWC | 4.1391 | 3.0316 | 0.1881 | ns |
| MBC | MacA | -1.6186 | 1.2925 | 0.2257 | ns |
| MBC | Sd | -73.6362 | 61.9962 | 0.2496 | ns |
| MAOC | (Intercept) | 19.4818 | 7.3063 | 0.0157 | * |
| MAOC | MBC | 0.0565 | 0.0162 | 0.0026 | ** |
| MAOC | MacA | 0.2887 | 0.095 | 0.007 | ** |
| MAOC | Sd | -18.8862 | 4.538 | 6e-04 | *** |
| MAOC | SWC | 0.5352 | 0.2244 | 0.0282 | * |
| MAOC | PB | -0.0465 | 0.0129 | 0.0021 | ** |
| TN | (Intercept) | 1.7786 | 1.3562 | 0.2053 | ns |
| TN | MBC | -0.0064 | 0.0025 | 0.0181 | * |
| TN | Sd | 0.2565 | 0.85 | 0.7661 | ns |
| TN | SWC | -0.0337 | 0.0328 | 0.318 | ns |
| TN | MAOC | 0.1212 | 0.0273 | 3e-04 | *** |

Table 10 Upload NCB database number

| ID | Accession number |
| --- | --- |
| ND1 | [SRR37096605](https://dataview.ncbi.nlm.nih.gov/object/SRR37096605) |
| ND2 | [SRR37096604](https://dataview.ncbi.nlm.nih.gov/object/SRR37096604) |
| ND3 | [SRR37096593](https://dataview.ncbi.nlm.nih.gov/object/SRR37096593) |
| ND4 | [SRR37096588](https://dataview.ncbi.nlm.nih.gov/object/SRR37096588) |
| ND5 | [SRR37096587](https://dataview.ncbi.nlm.nih.gov/object/SRR37096587) |
| ND6 | [SRR37096586](https://dataview.ncbi.nlm.nih.gov/object/SRR37096586) |
| SD1 | [SRR37096585](https://dataview.ncbi.nlm.nih.gov/object/SRR37096585) |
| SD2 | [SRR37096584](https://dataview.ncbi.nlm.nih.gov/object/SRR37096584) |
| SD3 | [SRR37096583](https://dataview.ncbi.nlm.nih.gov/object/SRR37096583) |
| SD4 | [SRR37096582](https://dataview.ncbi.nlm.nih.gov/object/SRR37096582) |
| SD5 | [SRR37096603](https://dataview.ncbi.nlm.nih.gov/object/SRR37096603) |
| SD6 | [SRR37096602](https://dataview.ncbi.nlm.nih.gov/object/SRR37096602) |
| MD1 | [SRR37096601](https://dataview.ncbi.nlm.nih.gov/object/SRR37096601) |
| MD2 | [SRR37096600](https://dataview.ncbi.nlm.nih.gov/object/SRR37096600) |
| MD3 | [SRR37096599](https://dataview.ncbi.nlm.nih.gov/object/SRR37096599) |
| MD4 | [SRR37096598](https://dataview.ncbi.nlm.nih.gov/object/SRR37096598) |
| MD5 | [SRR37096597](https://dataview.ncbi.nlm.nih.gov/object/SRR37096597) |
| MD6 | [SRR37096596](https://dataview.ncbi.nlm.nih.gov/object/SRR37096596) |
| HD1 | [SRR37096595](https://dataview.ncbi.nlm.nih.gov/object/SRR37096595) |
| HD2 | [SRR37096594](https://dataview.ncbi.nlm.nih.gov/object/SRR37096594) |
| HD3 | [SRR37096592](https://dataview.ncbi.nlm.nih.gov/object/SRR37096592) |
| HD4 | [SRR37096591](https://dataview.ncbi.nlm.nih.gov/object/SRR37096591) |
| HD5 | [SRR37096590](https://dataview.ncbi.nlm.nih.gov/object/SRR37096590) |
| HD6 | [SRR37096589](https://dataview.ncbi.nlm.nih.gov/object/SRR37096589) |
